# Remembering the Old Propensity Rules of the Electromagnetic Enhancement Mechanism of SERS: Reorientation of Pyridine on a Silver Electrode Induced by the Applied Potential

**DOI:** 10.1021/acs.jpcc.4c03084

**Published:** 2024-07-19

**Authors:** Samuel Valdivia, Francisco García-González, Daniel Aranda, Francisco J. Ávila Ferrer, Isabel López-Tocón, Juan Soto, Juan Carlos Otero

**Affiliations:** Andalucía Tech, Facultad de Ciencias, Departamento de Química Física, Universidad de Málaga, E-29071 Málaga, Spain

## Abstract

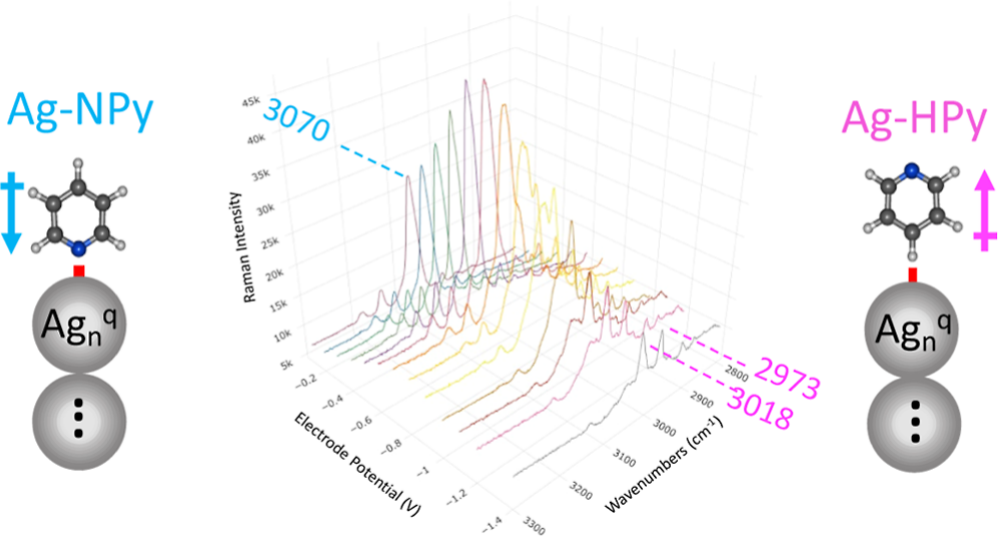

Electrochemical SERS of pyridine adsorbed on a silver electrode has been analyzed by comparing the spectra to the calculated normal Raman and resonance Raman intensities of model systems of pyridine bonded to linear silver clusters with different densities of charge through the nitrogen (Ag-NPy) or flipped through the hydrogen in the para-position (Ag-HPy). The changes observed in the ν(CH) region of the SERS have been investigated for the first time and related to a molecular reorientation at negative surface excess of charge of the metal in such a way that the ν(CH) bands with the highest (mode 2) and lowest (mode 13) wavenumber dominate this spectral region at positive or negative electrode potentials, respectively. The calculations support that the ν(CH) region is dominated by a specific vibration depending on pyridine orientation and suggest that both species coexist in the SERS recorded at negative potentials. This conclusion is supported by the SERS of centrosymmetric pyrazine which do not show this behavior and remembers the predictions from the old propensity rules of the so-called electromagnetic mechanism of SERS.

## Introduction

Electrified metal–molecule interfaces are key components in many technological devices, such as those involved in energy conversion and storage, the electrical transport through molecular junctions, or in classical electrochemistry. All these physical and chemical processes are very important but very difficult to control due to the lack of knowledge of the structure and properties of the components involved at the molecular level, namely, metals, molecules, ions, solvents, etc.

In the last 50 years, SERS^1,2^ has demonstrated to be a very powerful technique for observing fine details of these interfaces since the signal comes almost exclusively from molecules in direct contact with the metal. Therefore, it is possible to obtain very detailed information about metal–molecule hybrid systems by analyzing their spectra, but this becomes a formidable task in many cases, given that the SERS response of these complex systems is very often very difficult to unravel.

SERS is mainly characterized by the overall and the selective enhancement of the Raman scattering.^1,2^ However, the observed intensification is very dependent on the nature and morphology of the nanoplasmonic substrate, the nature of the molecule, the solvent and electrolyte, as well as many other experimental variables like the concentration of the involved chemical species, temperature, pH, laser excitation, and, mostly, the electric charges and fields present in any interface.^3^ These last two variables complicate even more the analysis of the results, although they can be controlled in electrochemical SERS experiments (EC-SERS) through the applied potential which allows us to tune and monitor the electrode processes.^3,4^ Applied bias modifies the entire electronic structure of the metal–molecule surface complex in the ground and excited electronic states, which affects the recorded spectra. As a result, the SERS of a molecule can show very different absolute and relative intensities depending on the particular conditions under which it has been recorded.

The standard methodology for analyzing SERS intensities consists of comparing the absolute and relative intensities of the bands of the normal Raman spectrum with those of respective SERS. The changes in the relative intensity of the bands of the molecule reflect the differences between the selection rules acting in Raman and SERS^2,5^ and are the main sources of information about molecules in interfaces. The metal is the main factor responsible for the overall enhancement mechanism through the plasmonic response of the nanostructured surface. This plasmonic physical mechanism is the most important contribution to SERS enhancement and was called “electromagnetic enhancement mechanism”.^1,2,6,7^

In the early days of SERS, general selection rules were used and extrapolated from IR or EELS experiments on surfaces.^2^ These rules were often called “propensity rules of the electromagnetic mechanism of SERS”^5,8−12^ which assume that the most intensified bands of the molecule should correspond to vibrations perpendicular to the metal due to the cancelation of the parallel component of the electromagnetic field in the surface. However, this is not strictly true for the visible light used as standard excitation in SERS and, moreover, the changes in the polarizability of a normal mode may have other components apart from those of the axis along which the molecule is vibrating. These simple rules assume that the molecule adopts a preferred orientation on the surface, which is determined by the chemical interaction between the metal and the molecule. These rules became very popular for years because they are general and easy to apply. Nevertheless, although some improvements were proposed such as restricting their use to particular symmetry groups or to exclusively analyze CH stretching bands,^5,9,10^ it was found that they did not work well in many cases, having been abandoned over time. For instance, the selective SERS enhancement of the out-of-plane vibrations of pyridazine is due to a distortion of this planar molecule in the excited charge transfer (CT) state and not to a flat adsorption on the surface.^13^

The spectroscopist intuited, and even forced in some analyses, the relationship between the preferred orientation of the adsorbate or the proximity of a part of a large molecule to the surface, with the selective intensification of specific SERS bands. Over time, the need for support for these hypotheses with quantitative estimates of the effect of these factors on the SERS of each system has gradually been accepted. As a result, many works published in SERS discuss the spectra based on theoretical calculations of absolute or relative intensities that are compared, with greater or lesser success, with the experimental results.^14^ These calculations require considering the supramolecular system involved in SERS through rather small,^15^ large,^16^ or very large^17^ models of metal clusters on which a molecule is linked at a more or less arbitrary local site. In some cases, it is necessary to consider the effect of applied potentials when discussing electrode experiments, although many times the effect of some other variables cannot be taken into account in the calculations.

These theoretical calculations are, generally, of the electronic structure of the metal–adsorbate system^14^ and would be the starting point for the quantitative estimation of the overall and/or selective SERS enhancement which requires knowing or assuming the participation of one or more of the intensification mechanisms that are nowadays accepted as relevant in SERS.^1,2,5^ If the theoretical predictions reproduced acceptably the observed experimental behavior, then it would be possible to confirm the suitability of the simplified metal–molecule model and the participation of the enhancement mechanisms considered.

The drastic simplification of modeling the interface in the calculations, the reliability of the electronic structure calculations themselves, and the unavoidable approximations made to estimate the effects of each mechanism in the Raman intensities determine the usefulness of the entire analysis, which can provide a comprehensive explanation of the experimental results or be totally useless, depending on the case.

SERS of pyridine (Py) recorded on silver at different electrode potentials is a good example of all of this. We have reported three kinds of contributions which are selected by the applied potential and characterized by the selective intensification of three different sets of bands^18^ which have been related to three different enhancement mechanisms dominating at positive, neutral, and negative surface excess of charge of the electrode, respectively. These changes in the relative intensities are observed in the medium region of the spectrum (200–1700 cm^–1^) and would originate from Py bonded to the metal through the lone electron pair of the nitrogen (Ag-NPy), giving a perpendicular orientation with respect to the surface.

In this work, the striking and never discussed changes observed in the CH stretching region of the SERS are discussed for the first time. It is proposed that they are due to the flipping of the molecule at very negative potentials in such a way that its dipole is reoriented, keeping the perpendicular orientation but with the hydrogen in para-position facing the metal surface (Ag-HPy).

## Methods

Details about EC-SERS experiments and the theoretical methodology for computing SERS intensities have been described elsewhere.^19^ Briefly, Raman spectra of pyridine and pyrazine (Sigma-Aldrich) have been recorded in a Renishaw InVia spectrometer working in macro conditions under 514.5 nm excitation. SERS spectra correspond to single scans with a 10 s exposure and 1 cm^–1^ spectral resolution. The Raman of a pure liquid and of a 1 M aqueous solution has been compared with the respective SERS of a 0.1 M water solution of the respective molecules (1 M KCl electrolyte) recorded on the rough surface of the working silver electrode of a three-electrode cell controlled by a potentiostat model 600E (CH Instruments Inc.) with a Pt counter electrode and a Ag/AgCl/KCl (sat.) reference electrode. The working electrode at −0.5 V was activated by subjecting it to seven 2 s pulses at +0.6 V in the absence of the molecule. Ultrapure water from a Milli-Q system was used for all solutions.

DFT and TD-DFT electronic structure calculations in the ground (S_0_) and the first 20 singlet excited states (S_*i*_, *i* = 1–20), respectively, have been carried out using the Gaussian09^20^ suit of programs with B3LYP^21−23^ and ωB97XD^24^ functionals and LanL2DZ^25−28^ and Def2TZVPP^29,30^ basis sets. Calculated vibrational wavenumbers and Raman intensities are similar regardless of the level of calculation (Table S1), and therefore, only B3LYP/LanL2DZ results will be used in the discussion.

**Figure 1:**
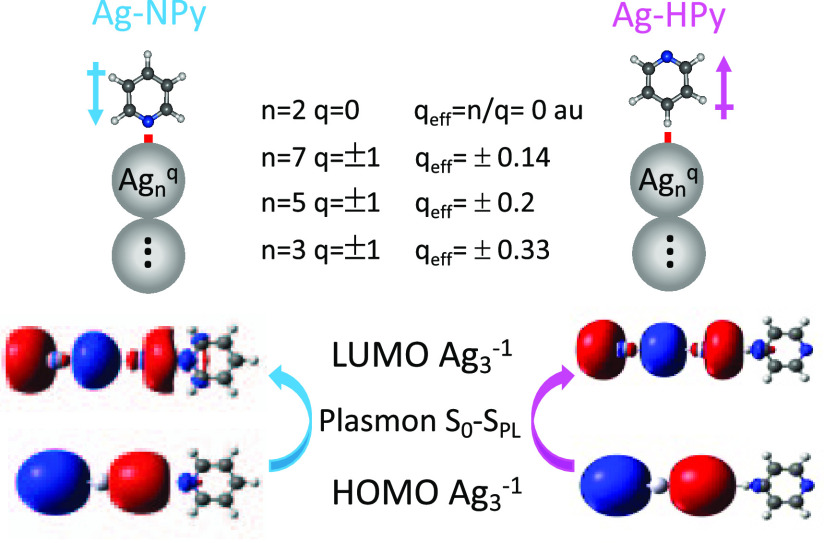
Structure of the linear [Ag_*n*_^*q*^-Py] models used in the calculations in which pyridine is bonded to the terminal silver atom through the nitrogen (Ag-NPy) or the hydrogen in the para-position (Ag-HPy). HOMO and LUMO orbitals of the respective [Ag_3_^–1^-Py] systems involved in the plasmonic (S_0_–S_PL_) electronic excitation of the silver clusters are also shown.

Calculations have been carried out for isolated pyridine and bonded to a set of linear stick-like Ag_*n*_^*q*^ clusters [Ag_*n*_^*q*^–Py] of different lengths (n = 2 and 3, 5, and 7) and charges (*q* = 0 and ±1, respectively) (Figure 1).^31^ These systems allow us to define the density of charge *q*_eff_ = *q*/*n* for each Ag_*n*_^*q*^ cluster that mimics the effect of the electrode potential by changing the surface excess of charge of the silver atom on the surface to which Py is attached. The molecule is bonded to the terminal silver atom of the clusters, simulating a perpendicular adsorption on the surface through the nitrogen (Ag-NPy) or the hydrogen in the para-position (Ag-HPy) (Figure 1). Geometry optimizations have been carried out keeping the structures of the silver clusters and of metal–pyridine complexes linear (*C*_2*v*_ symmetry). For each [Ag_*n*_^*q*^–Py] system, Raman activities in the ground electronic state have been computed, as well as the intensities in preresonance conditions with a single selected excited state or using a multistate approach at fixed 514.5 nm excitation (see details in the Supporting Information).

## Results and Discussion

### Experimental EC-SERS Results of Pyridine

**Figure 2:**
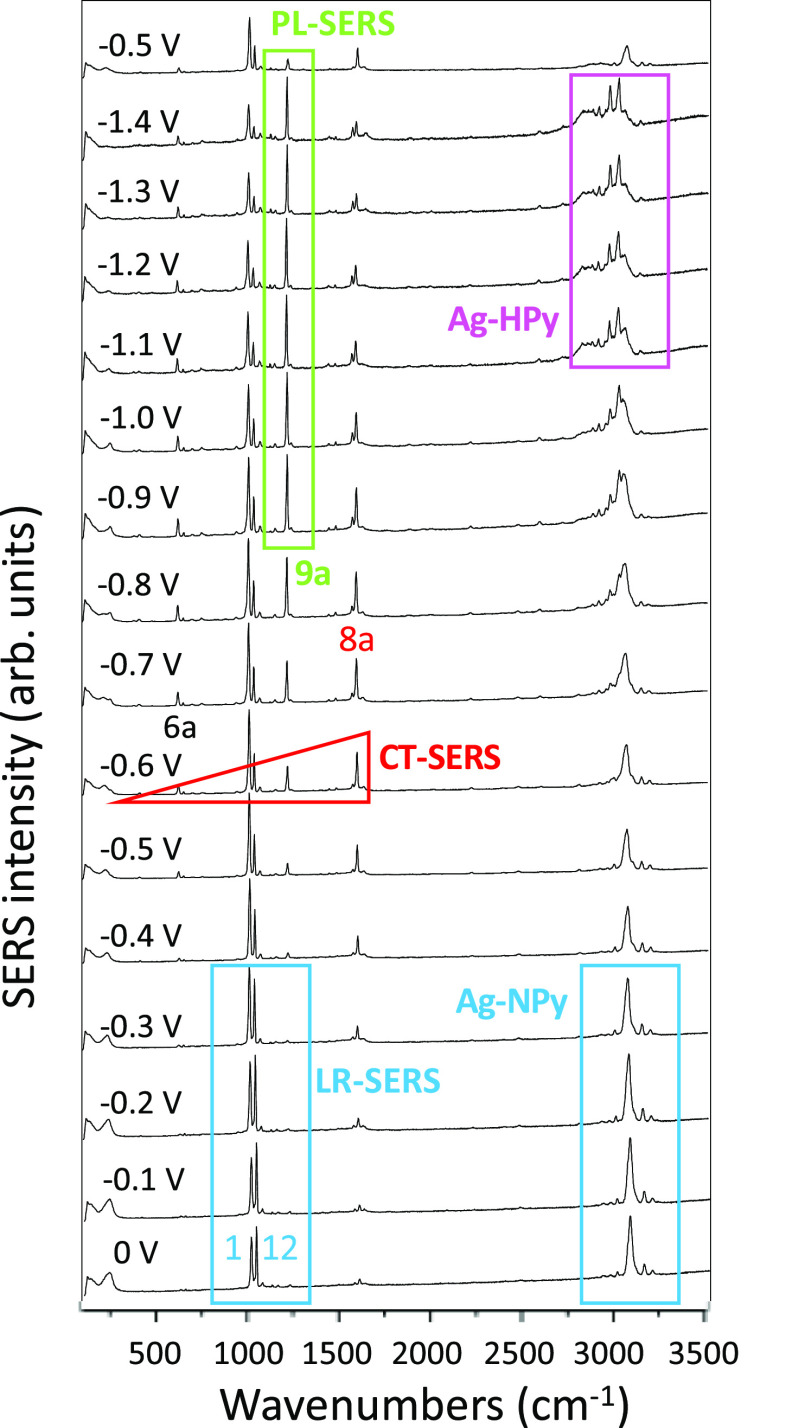
SERS spectra of pyridine recorded at different electrode potentials (514.5 nm excitation). Relative intensities are normalized to the strongest band.

This work is focused on the analysis of the changes in the relative enhancement of the bands observed in EC-SERS due to the applied bias. Figure 2 shows the SERS of Py recorded between 0 and −1.4 V and back to −0.5 V. The intensities of the 200–1800 cm^–1^ region have already been discussed in previous works,^18,32^ showing three different types of spectra which are characterized by the specific enhancement of particular bands. Vibrations 1;ν(ring) and 12;δ(ring) appear at ca. 1000 cm^–1^ and dominate the SERS recorded at positive potentials. These spectra are reminiscent of the normal Raman of the pure liquid or the aqueous solution of Py as well as of its SERS on silver hydrosol.^33^ They do not show remarkable selective enhancement of other bands and would be enhanced through a general plasmonic mechanism; therefore, they were called long-range SERS (LR-SERS, Figure 2). This would imply that the interface modifies slightly the polarizability of the normal modes of the free molecule and, therefore, there is not any resonant process involving the adsorbate.

From −0.5 to −0.9 V, the selective enhancement of the bands corresponding to modes 6a;δ(ring), 1;ν(ring), 9a;δ(CH), and, mainly, 8a;ν(ring) which is recorded at ca. 1600 cm^–1^, can be appreciated, yielding a triangular shape.^13,14,17,18,31^ We have shown that this very characteristic behavior is due to a resonant Raman process involving the metal-to-molecule CT process in benzene-like systems or azabenzenes.^32^ The electrode potential tunes the energies of the CT states^31^ in such a way that they match the energy of the laser photon in this range of potentials (CT-SERS). The absolute intensity of the SERS decays at more negative potentials than −0.6 V due to two effects, the system becomes out of resonance, and the molecular coverage of the electrode is reduced given that it is generally assumed that Py is adsorbed by electron donation from the lone pair of the nitrogen to vacant orbitals of the metal (Ag-NPy), which is favored at positive potentials.

However, the band assigned to vibration 9a;δ(CH) at 1200 cm^–1^ becomes the strongest one at more negative potentials than −0.9 V. It has been demonstrated that this selective enhancement is related to the large amplitude clapping of the *ortho*-CH bonds inside the bulky negative density of charge of the surface when the laser excites plasmon-like states of the very negative charged silver clusters (PL-SERS).^18^ These three kinds of SERS correspond to perpendicular Ag-NPy complexes, and the CT and PL contributions at medium and negative potentials have been confirmed through theoretical calculations of the respective resonant Raman intensities.^18,31,32^

**Figure 3:**
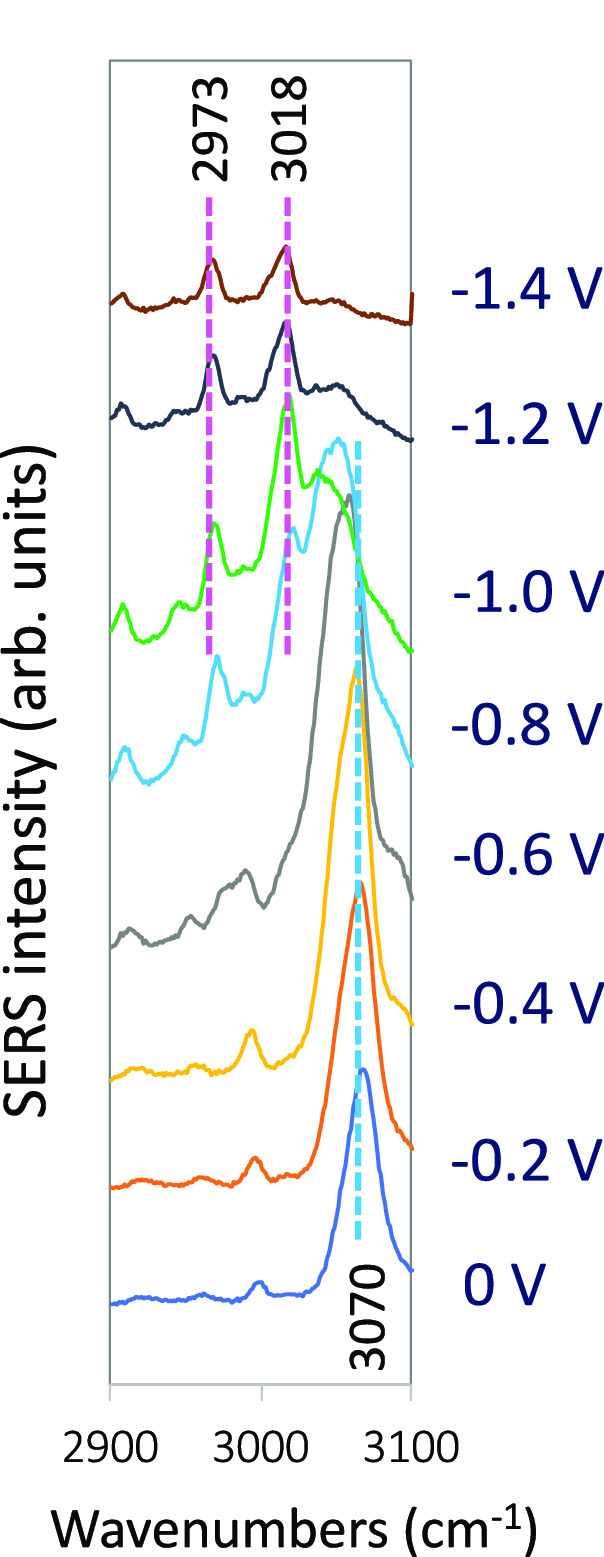
ν(CH) region of the SERS spectra of pyridine recorded at different electrode potentials (514.5 nm excitation).

However, much less attention has been devoted to the high wavenumber region of the EC-SERS. A single strong ν(CH) stretching band at ca. 3070 cm^–1^ dominates this zone of the SERS at 0 V (Figures 1 and 3), with similar intensity to those of vibrations 1 and 12 as occurs in the normal Raman spectrum of Py, so it could be considered to belong to the set of vibrations corresponding to the LR-SERS type of spectra of the Ag-NPy complex. The relative intensity of this line and that of mode 12 decrease continuously when the electrode potential is made more negative since vibrations ν(CH) and 12 should be weakly involved in the chromophore of the CT or PL resonances.^18^

This band almost disappears in the SERS recorded at potentials more negative than −1.2 V, while two new bands measured at ca. 2973 and 3018 cm^–1^ can already be observed at −0.8 or −0.9 V, respectively (Figure 2). The relative intensity of these two bands increases continuously up to −1.4 V. The band at 3018 cm^–1^ becomes stronger than vibration 12 at −1.1 V, and its intensity is 1/3 of that of mode 1 and 1/4 of vibration 9a, while it is as intense as mode 1 and reaches 1/2 of the 9a band in the spectrum at −1.4 V. The lower wavenumber band of this couple (2973 cm^–1^) increases its relative intensity as the potential becomes more negative (Figure 3).

### Calculated Normal Raman Spectra of Ag-NPy Systems

B3LYP/LanL2DZ calculations of the Raman intensities were carried out in order to account for the behavior of the ν(CH) SERS bands. The three ν(CH);A_1_ fundamentals of isolated Py (modes 13, 20a, and 2) are calculated at 3241, 3211, and 3199 cm^–1^, respectively, and the two B_2_ modes (7b and 20b) appear intercalated at 3228 and 3201 cm^–1^, respectively. The atomic displacements of each vibration can be seen in the movie Nomal modes (see the Supporting Information), where they can be compared with the results obtained for the [Ag_3_^–1^-NPy] complex. Apparently, all the wavenumbers are red-shifted about −10 cm^–1^ in the complex, but a reversed order of the respective fundamentals is detected from the shape of the vibrations, in such a way that the all-in-phase 2;ν(CH);A_1_ vibration shows the lowest wavenumber in Py (3199 cm^–1^) and the highest value (3232 cm^–1^) in [Ag_3_^–1^-NPy]. It is to be expected that this totally symmetric fundamental should correspond to the strong experimental SERS band of 3070 cm^–1^ recorded at positive potentials.

**Figure 4:**
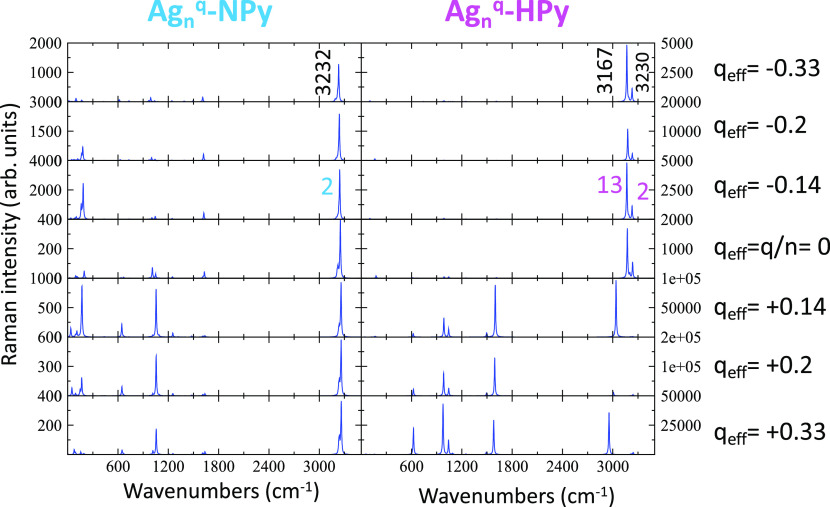
Dependence of the B3LYP/LanL2DZ calculated normal Raman spectra of Ag-NPy and Ag-HPy complexes on the density of charge of the silver clusters (*q*_eff_).

Concerning Raman intensities, the calculated spectra of the [Ag_*n*_^*q*^-NPy] series of complexes in the ground electronic state (Figure 4) predict the appearance of a single ν(CH) strong band for all of them, in agreement with the SERS recorded at positive/medium potentials. This band is the all-in-phase 2;ν(CH) stretching of a higher wavenumber (3232 cm^–1^). According to these spectra, this band would gain relative intensity when the negative charge of the metal increases, but this behavior is not observed given that it almost disappears at potentials more negative than −1.0 V because this vibration is not involved in the CT or PL resonant processes which are dominating the spectra at negative potentials.

Summarizing, the properties of the N-bonded complexes in the ground or excited electronic states explain the observation of a single CH stretching band in the SERS recorded at positive or neutral metal surfaces but are unable to account for the appearance of a pair of new ν(CH) bands at negative potentials.

### Adsorption Energy of Ag-NPy and Ag-HPy Complexes

**Figure 5:**
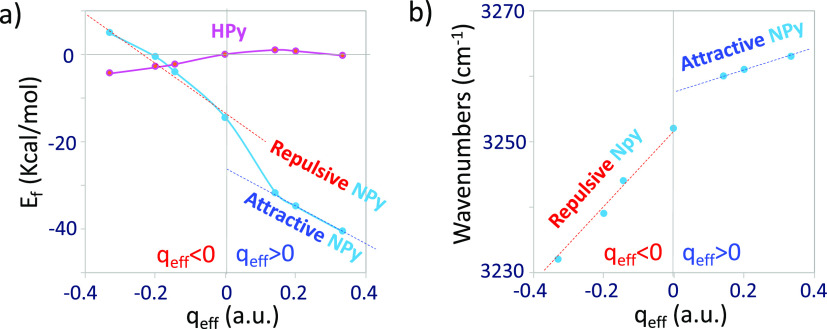
Effect of the density of charge of the silver clusters (*q*_eff_) on the B3LYP/LanL2DZ calculated values of (a) adsorption energies (*E*_f_) of the Ag-NPy and Ag-HPy complexes and (b) wavenumber of the highest 2;ν(CH) mode of the Ag-NPy system.

Figure 5a shows the dependence of the B3LYP/LanL2DZ calculated energies of formation (*E*_f_) of the Ag-NPy metal–molecule complexes [*E*_f_ = *E*_Ag-Py_ – (*E*_Ag_ – *E*_Py_)] on *q*_eff_. *q*_eff_ is an atomistic parameter and models the tuning of the macroscopic surface excess of charge of the electrode by the applied bias. Its amount and sign modulate the strength of the Ag-NPy complex in the ground state.^31^ As expected, they are more stable as the metal has a more positive density of charge, but the dependence is not linear, showing two different behaviors at positive or negative *q*_eff_ values. This result reflects the dual electronic structure of the metal–molecule surface complexes depending on the sign of the charge of the metal, giving two different types of systems with attractive (*q*_eff_ > 0 au) and repulsive (*q*_eff_ < 0 au) character.^34^ Besides the chemical Ag–N bond, the direction of the dipole moment of pyridine in the Ag-NPy complexes (Figure 1) favors the adsorption in the case of positive charged silver atoms (attractive) with *E*_f_ ranging between −31.7 and −40.7 kcal/mol, while it only amounts to values from −4.1 to +4.9 kcal/mol in repulsive complexes with *q*_eff_ < 0.

The chemical Ag–N bonding between the nitrogen and silver disappears in the flipped Ag-HPy systems in which the much weaker charge(metal)–dipole(pyridine) interaction is responsible for the existence of this species and the behavior shown by the corresponding *E*_f_ (Figure 5a).

Ag-HPy complexes have zero or near-zero *E*_f_ values at *q*_eff_ = 0 or *q*_eff_ > 0 au, being much less stable than the corresponding Ag-NPy complexes. On the contrary, there is an attractive interaction between the dipole of pyridine and the negative charge of the metal at *q*_eff_ < 0 au (Figure 1). In this case, Ag-NPy and Ag-HPy have similar *E*_f_ values, and hence, both complexes could coexist when the metal has a negative charge. In the extreme case of *q*_eff_ = −0.33 au, *E*_f_ amounts to −4.2 kcal/mol for the Ag-HPy system which is 9.1 kcal/mol more stable than the corresponding N-bonded complex. Therefore, theoretical calculations predict the presence of flipped pyridine at more negative values of the potential of zero charge of the electrode (*E*_pzc_), while the existence of [Ag_*n*_^*q*^–HPy] would be ruled out at *q*_eff_ ≥ 0 au given that Ag-NPy systems should be much more stable.

### Calculated Normal Raman Spectra for Ag-HPy Systems

The same type of calculations has been carried out for the [Ag_*n*_^*q*^–HPy] series, but the discussion will be focused on negative *q*_eff_, where *E*_f_ of these complexes competes with or is more stable than that of Ag-NPy. Vibrational wavenumbers and shape of the ν(CH) normal modes for both types of complexes with *q*_eff_ = −0.33 au are compared in the Normal modes file of the Supporting Information. Although the results appear to be similar, two differences can be seen. A smooth mode rotation can be appreciated, causing changes in the atomic displacements and wavenumbers of both complexes centered in the low wavenumber mode 13;ν(CH) (3167 cm^–1^), which shows the largest and very significant shift of −23 cm^–1^ with respect to the same vibration of Ag-NPy. Both differences are related to the very large amplitude of the hydrogen in the para-position that vibration 13 shows inside the large density of negative charge of the silver cluster in [Ag_3_^–1^-HPy] (see the HOMO and LUMO orbitals in Figure 1).

**Figure 6:**
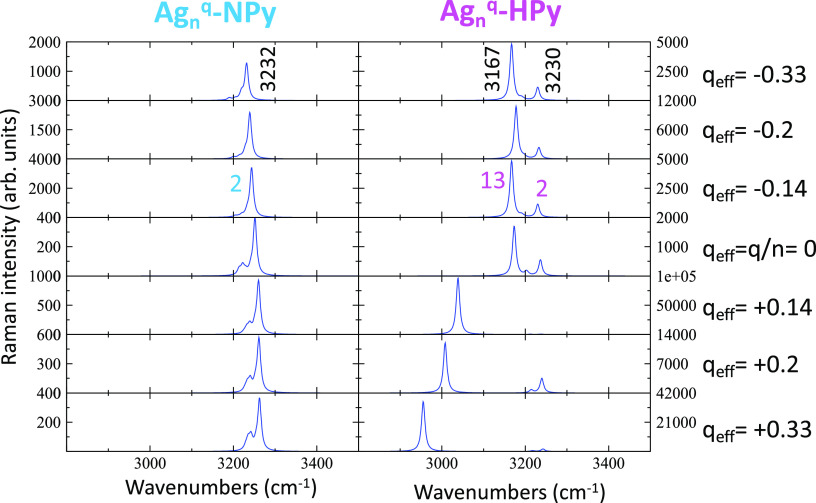
Effect of the density of charge of the silver clusters (*q*_eff_) on the ν(CH) region of the B3LYP/LanL2DZ-calculated normal Raman spectra of Ag-NPy and Ag-HPy complexes.

Calculated normal Raman spectra of both complexes are compared in Figure 4. Ag-HPy spectra at *q*_eff_ < 0 au are much more intense than those of Ag-NPy, and the all-in-phase 2;ν(CH) band of the Ag-HPy complex (3230 cm^–1^) is now predicted to be much weaker. Opposite, the intensity of the mode 13 at 3167 cm^–1^ dominates the Ag-HPy calculated spectra, while the intensities of the remaining bands should be almost negligible. These results suggest an equilibrium between both species at very negative potentials and agree with the experimental behavior of SERS recorded at potentials more negative than *E*_pzc_ where the strongest ν(CH) band recorded at positive potentials (Ag-NPy) is replaced by a couple of bands (Ag-HPy), but the relative intensities of the pair of ν(CH) vibrations are very different and in reverse order of the experimental ones (see Figure 6).

### Calculated Resonant Raman Spectra of [Ag_3_^–1^–Py] Complexes

**Figure 7:**
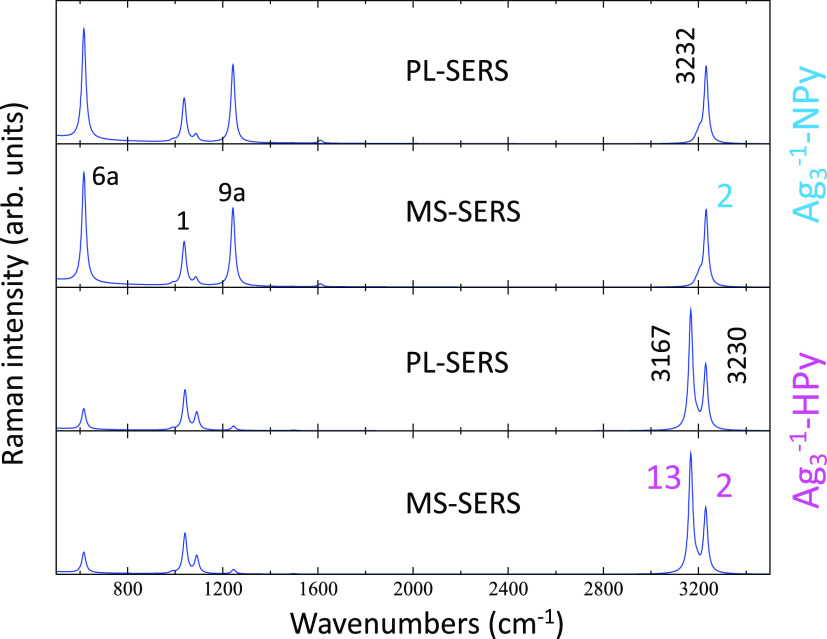
Raman spectra of the [Ag_3_^–1^-NPy] and [Ag_3_^–1^-HPy] complexes calculated from B3LYP/LanL2DZ results in preresonance with the plasmonic excited state (PL-SERS) or using a multistate approach (MS-SERS) (see the Supporting Information).

Previous results refer to normal Raman spectra, but preresonance intensities of Ag-NPy and Ag-HPy complexes with *q*_eff_ = −0.33 au have also been calculated under two approaches: (i) considering a single singlet excited state located inside the silver cluster showing the largest oscillator strength (called plasmonic PL state, PL-SERS), which corresponds to the respective HOMO–LUMO transition (see Figure 1) and (ii) using the multistate approach (MS-SERS) where the weighted contributions of the first 20 singlet excited states to the resonant Raman intensities under 514.5 nm excitation are taken into account (see the Supporting Information for details). Figure 7 shows that PL and MS-SERS are identical, given that the PL excitation is so strong as to dominate all other contributions. The energy of this PL state in both complexes is 2.44 and 2.47 eV, respectively, and the corresponding oscillator strength amounts to 1.99 and 1.25, respectively. In the case of [Ag_3_^–1^-NPy], a single strong 2;ν(CH);A_1_ band is predicted (Figure 5) as it happened in the respective calculated normal Raman spectrum, but its relative intensity is now drastically reduced with respect to the bands recorded in the 200–1600 cm^–1^ region.

The appearance of two ν(CH) bands is predicted again for [Ag_3_^–1^–HPy], but their intensities are more balanced than those in the calculated normal Raman spectrum. The intensity of the low wavenumber 13;ν(CH) band is now reduced when compared to the all-in-phase mode 2, being in better agreement with the experiments. Furthermore, CH bands of this complex continue to be much more intense than those calculated in the medium region of the spectra. Therefore, the combination of the calculated results of normal and resonance Raman intensities indicates that the expected spectrum for the Ag-HPy system recorded at negative potentials should be characterized by only two strong CH stretching bands, while the intensity of the remaining vibrations would be almost negligible.

### Experimental EC-SERS Results of Pyrazine

**Figure 8:**
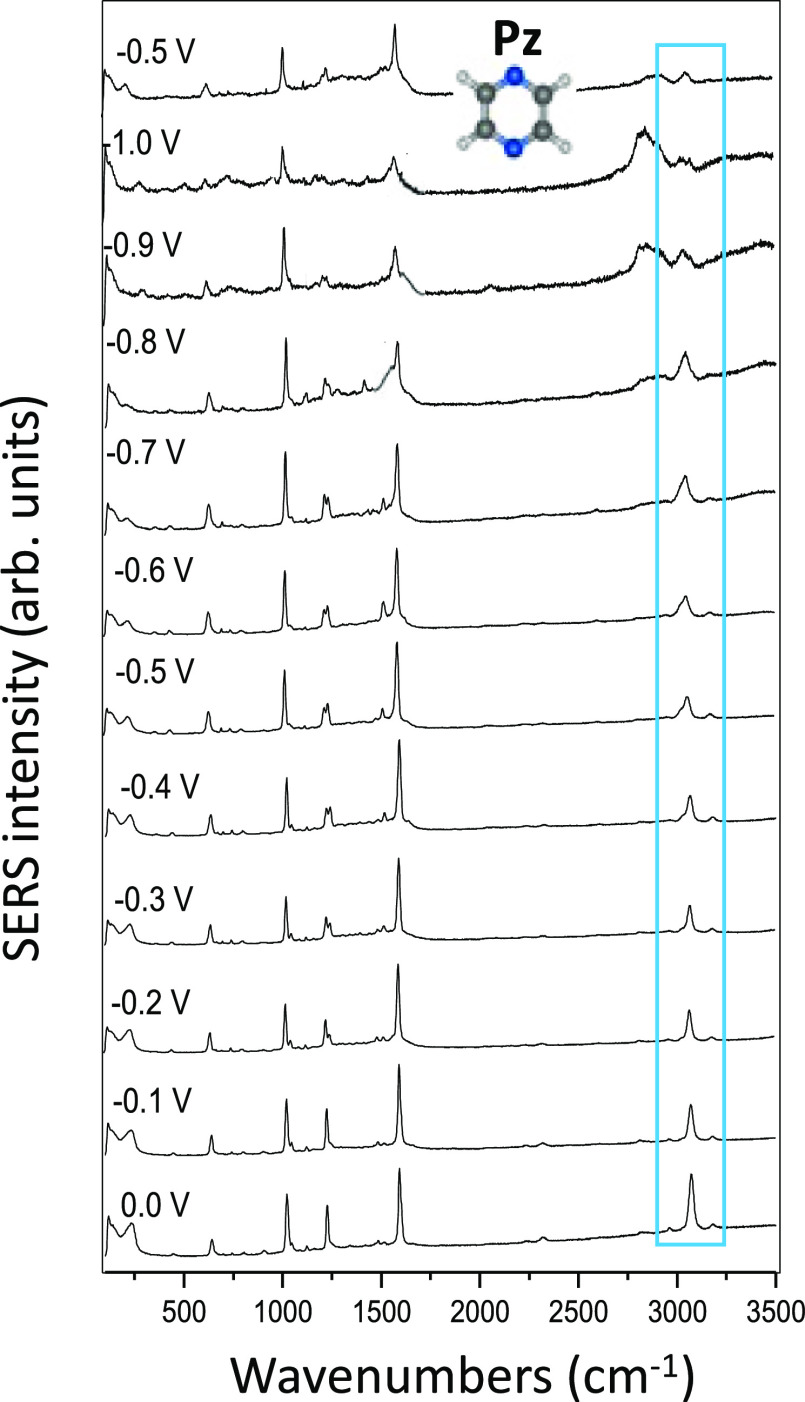
SERS spectra of pyrazine recorded at different electrode potentials (514.5 nm excitation). Relative intensities are normalized to the strongest band.

The changes in the high wavenumber region have been related to the flipping of pyridine-adsorbed perpendicular to the electrode, giving a realignment of its dipole along the normal direction of the surface at positive or negative potentials, respectively. Pyrazine(1,4-diazine) is a centrosymmetric molecule, and therefore, their SERS spectra should not exhibit this behavior, given that a reorientation would produce the same initial structure. EC-SERS of Pz recorded between 0.0 and −1.0 V and back to −0.5 V are drawn in Figure 8.

The absence of a dipole makes the adsorption of Pz on silver less stable than in the case of Py, and very weak spectra can be registered at potentials more negative than −1.0 V in this case. As can be seen, a single ν(CH) band is recorded in all the spectra, while the couple of bands related to Ag-HPy could already be detected at −0.8 or −0.9 V. A very broad band recorded at ca. 2800 cm^–1^ is observed in the SERS of Py and Pz and should be originated by decomposition products.

### Dual Electronic Structure of Metal–Molecule Surface Systems

The dual behavior shown by the properties of metal–molecule surface complexes has already been discussed in previous works and is more evident in the case of anionic adsorbates like isonicotinate,^34^ cyanide,^35^ or cyanobenzoate,^36^ given that the Coulombic interaction between charges [charge(metal)–charge(anion)] should be stronger than for dipolar [charge(metal)–dipole(pyridine)] or neutral [charge(metal)–neutral(pyrazine)] molecules. As a result, the sign of the charge of the metal selects two types of surface complexes of different nature for the same adsorbate which are characterized by the differentiated sensitivity of their properties (adsorption energy,^34−36^ vibrational wavenumbers,^35,36^ and type of forward or reverse metal–molecule CT excited states^34^) on the applied potential. In the case of charged molecules, usually organic anions, the electrostatic interaction with the metal is so strong that the attractive and repulsive complexes are of different nature and were related to chemisorbed and physisorbed species, respectively.^34,35^ The greater the attractive interaction, the flatter the dependencies of the respective properties are in such a way that the potential is almost unable to modify the overall electronic structure of the very strong surface complexes. Therefore, *E*_f_ or the energies of the CT states remains almost unaltered. On the contrary, the sensitivity to the potential becomes more acute when the repulsive interaction between the metal and the molecule is stronger.

The *E*_f_ vs *q*_eff_ plots of the Ag-NPy and Ag-HPy systems in Figure 5a are reflecting the dual behavior in the case of this neutral adsorbate. Vibrational wavenumbers are also very sensitive to the applied potential and, therefore, to changes in the electronic structure of metal–molecule hybrid systems as ν(CN^–^) and ν_s_(CO_2_^–^) stretching vibrations show in the SERS of cyanide^35^ or cyanobenzoate^36^ anions, respectively. In both cases, these vibrations are directly involved in the adsorption to the metal, which explains the sensitivity of their wavenumbers to the applied bias. The same occurs to the calculated wavenumbers of the all-in-phase 2;ν(CH) vibration of the Ag-NPy complex (Figure 5b), although these bonds are not in direct contact with the metal. The respective wavenumbers of repulsive complexes at negative *q*_eff_ have a larger slope, which is greatly reduced when the metal has positive charge. Unfortunately, the experimental results do not allow us to confirm the theoretical predictions given that sharp ν(CH) bands can be only observed in a short range of potentials, but the SERS wavenumbers of the most characteristic vibrations of pyridine recorded in the 200–1700 cm^–1^ region also seem to show this dual dependence.^37^

## Conclusions

The ν(CH) region of the EC-SERS spectra of pyridine shows a single band when the spectra are recorded at potentials more positive than the *E*_pzc_ of the silver electrode, being replaced by a pair of new bands at more negative potentials, while the wavenumbers of the remaining vibrations can be correlated along the entire range of potentials.

Raman intensities based on DFT calculations of linear [Ag_*n*_^*q*^-Py] complexes with different densities of charge (*q*_eff_ = *q*/*n*) are able to explain the experimental results on the basis of a reorientation of a fraction of adsorbed molecules. The calculated Raman spectra of complexes with pyridine bonded to silver through the nitrogen (Ag-NPy) predict the appearance of a single ν(CH) stretching band with the highest wavenumber and assigned to all-in-phase mode 2. The *para*-hydrogen of flipped pyridine (Ag-HPy) faces the metal with negative charge, and the respective calculated spectra predict in turn that this same vibration and the fundamental of lowest wavenumber [13;ν(CH)] should be the strongest ones. Calculations also predict that the Ag-HPy complex is the most stable at negative electrode potentials and that the Raman spectrum of this species should be almost exclusively characterized by this pair of ν(CH) bands. The combined experimental and computational study here presented reveals a reorientation induced by the electrode potential being undetectable by analyzing the 200–1700 cm^–1^ medium region of SERS which highlights the importance of analyzing the ν(CH) zone often neglected in most SERS studies.

The silver–pyridine surface complex is very weak at very negative potentials, as demonstrated by the weak intensities of the SERS scattering from the few adsorbed molecules. Under these circumstances, the metal–molecule chemical interaction does not significantly perturb the properties of the pyridine, and the most enhanced vibrations seem to fulfill the propensity rules of the electromagnetic mechanism applied to both types of complexes. Vibration 9a;δ(CH) dominates the SERS in the case of Ag-NPy due to the proximity of the CH in the ortho-position to the surface as well as to the large amplitude motions of the deformations of these bonds inside the large and diffuse negative density of charge of the surface. The flipping of the molecule reverses the orientation of pyridine, and the *para*-CH is now the closest bond to silver, being the cause of the enhancement of the 13;ν(CH) fundamental, which is precisely the one that shows the largest amplitude of this bond. The difference between them is that the enhancement of 9a is related to a resonant process involving a plasmon-like excitation^18^ inside the metal and that the enhancement of mode 13;ν(CH) of the flipped structure can be related to both the ground and the excited plasmonic states.

EC-SERS of pyridine is a good example of the complexity of understanding the experimental results of this kind of spectra. This molecule was used by Fleischmann et al. to record the first SERS 50 years ago,^38^ and since then, its spectrum has been the subject of a lot of works. Despite this, SERS of pyridine continues to provide new and valuable information about metal–molecule charged interfaces.

## Supporting Information Available

Additional computational details and calculated wavenumbers and Raman intensities of [Ag_3_^–1^-NPy] and [Ag_3_^–1^-HPy] at several levels of theory (PDF)
Normal modes (MPG)

## Supplementary Material

jp4c03084_si_001.pdf

jp4c03084_si_002.mpg
