# Supplementary material for: Remembering the Old Propensity Rules of the Electromagnetic Enhancement Mechanism of SERS: Reorientation of Pyridine on a Silver Electrode Induced by the Applied Potential
Source: J Phys Chem C Nanomater Interfaces. 2024 Jul 19;128(30):12566–74. doi: 10.1021/acs.jpcc.4c03084 (PMC11299182; doi:10.1021/acs.jpcc.4c03084)
Supplement: Supplementary file 1 — jp4c03084_si_001.pdf [file jp4c03084_si_001.pdf]

SUPPLEMENTARY INFORMATION

Remembering the Old Propensity Rules of the Electromagnetic Enhancement Mechanism of SERS: Reorientation of Pyridine on Silver Electrode Induced by the Applied Potential

Samuel Valdivia[1#], Francisco García-González[1#], Daniel Aranda[1], Francisco J. Ávila Ferrer[1], Isabel López-Tocón[1], Juan Soto and Juan Carlos Otero[1*]

Address:

[1]Andalucía Tech, Facultad de Ciencias, Departamento de Química Física, Universidad de Málaga, E-29071 Málaga, Spain

***Corresponding author:** Juan Carlos Otero – Email: jc_otero@uma.es

# These authors contribute equally to this work.

CALCULATION DETAILS

RR intensities

Preresonance Raman (RR) intensities have been calculated by using the methodology by A. Jarzecki[1] following two different ways:

Single-state approximation:

Only a single S_g-S_e resonant excitation involving the ground (S_g) and the selected excited state (S_e) is taken into account which, in this case, corresponds to the state with the strongest oscillator strenght (Plasmon-like state, PL). RR intensities are calculated by assuming short-time propagation of the ground state wave function on the single excited state as well as the gradient approximation, i.e., the excited-state displacements with respect to the ground state geometry are proportional to the gradient of the excited state potential energy surface in the Franck-Condon region. The intensities for the k-th mode can be estimated from the equation:

$$I_{k,FC}^{g\rightarrow e} \propto (v_o - v_k)^3 v_o \left(\frac{v_k}{\mu_k}\right)\left(\frac{\partial E_e}{\partial Q_k}\right)^2 \qquad (1)$$

where v_o is the excitation energy, v_k is the vibrational wavenumber of the k-th mode in the electronic ground state S_g, μ_k is the corresponding effective mass and $\left(\frac{\partial E_e}{\partial Q_k}\right)$ is the gradient with respect to the respective normal mode Q_k:

$$\left(\frac{\partial E_e}{\partial Q_k}\right) = \boldsymbol{f}\boldsymbol{M}^{-\frac{1}{2}}\boldsymbol{L}_k \qquad (2)$$

which is obtained[2,3] from the Cartesian molecular forces $\boldsymbol{f}$ calculated in the excited state at the Franck-Condon geometry, $\boldsymbol{M}$ is the diagonal matrix of atomic masses and $\boldsymbol{L}_k$ is the normal mode matrix element associated with the k-th fundamental.

Multi-states approximation:

The limitation of the single-state gradient formulation described above is extended to several states by introducing the weighted-gradient approximation,[1] where each computed excited-state gradient is weighted by the appropriate $W_{\Gamma,e}$ factor at the amplitude level as shown in Eq. 3:

$$I_{k,FC}^{g\to e} \propto (v_o - v_k)^3 v_o \left(\frac{v_k}{\mu_k}\right) \Sigma_e \left| \left(\frac{\partial E_e}{\partial Q_k}\right)^2 W_{\Gamma,e} \right|^2 \tag{3}$$

with

$$W_{\Gamma,e} = \frac{|\mu_e^g|^2}{N} \left[\frac{\Gamma_e^2}{\Gamma_e^2 + \left(v_o - (v^{e,k} - v^{g,j})\right)^2} \right] \tag{4}$$

where $|\mu_e^g|$ is the dipole moment integral, Γ_e^2 the empirically determined energy offset (damping parameter) and $\left(v^{e,k} - v^{g,j}\right)$ is the energy of the electronic transition.

The normalization constant N is obtained so a full spectrum of computed weighting factors sum up to unity:

$$N = \left[\sum_e \left\{ \left|\mu_e^g\right|^2 \left[\frac{\Gamma_e^2}{\Gamma_e^2 + \left(v_o - \left(v^{e,k} - v^{g,i}\right)\right)^2} \right] \right\}^2 \right]^{1/2} \tag{5}$$

Table S1.- Vibrational wavenumbers and Raman intensities of the ν(CH) vibrations of [Ag_3^{-1}-NPy] and [Ag_3^{-1}-HPy] complexes calculated at several levels of theory.

B3LYP/LANL2DZ							
		Ag_3^--NPy				Ag_3^--HPy	
		Wavenumber	Raman Int.			Wavenumber	Raman Int.
	A1	3190.0	73.6		A1	3167.1	4822.5
	B2	3196.8	6.3		B2	3190.7	140.5
	A1	3204.1	48.9		A1	3194.1	43.6
	B2	3219.3	235.2		B2	3224.3	0.7
	A1	3231.6	1254.9		A1	3229.8	1109.1
**B3LYP/DEF2TZVPP**							
		Ag_3^--NPy				Ag_3^--HPy	
						Wavenumber	Raman Int.
					A1	3133.9	5083.4
		dissociates			B2	3137.1	173.3
					A1	3141.3	331.5
					B2	3188.2	0.1
					A1	3189.0	1050.0
**WB97XD/LANL2DZ**							
		Ag_3^--NPy				Ag_3^--HPy	
		Wavenumber	Raman Int.			Wavenumber	Raman Int.
	A1	3235.9	89.8		A1	3215.5	4173.3
	B2	3237.1	0.5		B2	3228.2	137.7
	A1	3245.8	29.2		A1	3232.2	53.6
	B2	3266.5	180.1		B2	3269.9	1.2
	A1	3278.2	661.5		A1	3275.4	788.3
**WB97XD/DEF2TZVPP**							
		Ag_3^--NPy				Ag_3^--HPy	
		Wavenumber	Raman Int.			Wavenumber	Raman Int.
	B2	3177.5	0.4		B2	3167.5	157.3
	A1	3178.7	196.8		A1	3169.2	1765.7
	A1	3202.8	61.2		A1	3174.3	2519.8
	B2	3224.3	184.8		B2	3228.1	2.7
	A1	3231.2	721.9		A1	3229.5	645.7

References

1. A. A. Jarzecki, *J. Phys. Chem.* A 2009, **113**, 2926 and references therein.

2. F. Zerbetto and M. Z. Zgierski, *J. Chem. Phys.* 1994, **101**, 1842.

3. F. Avila, J. Soto, J. F. Arenas, J. A. Rodríguez, D. Peláez and J. C. Otero, *J. Phys. Chem. C*, 2009, **113**, 105.
